# 

**Published:** 1878-03

**Authors:** 


### TABLE OF CONTENTS.

ORIGINAL COMMUNICATIONS.

Puerperal Eclampsia. *By J. W. Griggs, M. D., West Point, Ga.* 705
Quinine—Inaugural Thesis. *By Thomas Northern*.................... 711

REPORTS OF SOCIETIES.

Atlanta Academy of Medicine.................... 714

Montreal Medico-Chirurgical Society.................... 715

Minutes of the Pharmaceutical Meeting.................... 717

New York Therapeutical Society.................... 719

Baltimore Medical and Surgical Society.................... 723

Chicago Medical Society.................... 725

SELECTIONS.

Pennsylvania Hospital—Clinic of Dr. J. M. DaCosta.................... 731
Good Samaritan Hospital Medical Clinic.................... 738
A Contribution to the Study of Hog Cholera.................... 743
The Antecedent Treatment of those Exposed to Zymotic Diseases.... 750

EDITORIAL.

Unethical Advertisements.................... 756

Atlanta Medical College.................... 757

Medical Association of Georgia.................... 758

American Pharmaceutical Association.................... 759

Georgia Pharmaceutical Association.................... 759

Vaccination.................... 760

To our Subscribers.................... 760

Pamphlets Received.................... 760

Editorial Correspondence.................... 761

EXCERPTA.

Intra-Uterine Injection of Hot Water in Post Partum Hemorrhage.... 763
Sciatic Neuralgia.................... 763

